# Application of unmanned aerial systems for high throughput phenotyping of large wheat breeding nurseries

**DOI:** 10.1186/s13007-016-0134-6

**Published:** 2016-06-24

**Authors:** Atena Haghighattalab, Lorena González Pérez, Suchismita Mondal, Daljit Singh, Dale Schinstock, Jessica Rutkoski, Ivan Ortiz-Monasterio, Ravi Prakash Singh, Douglas Goodin, Jesse Poland

**Affiliations:** Department of Geography, Kansas State University, Manhattan, KS 66506 USA; International Maize and Wheat Improvement Center (CIMMYT), Int. Apdo., Postal 6-641, 06600 Mexico, D.F. Mexico; Interdepartmental Genetics Program, Kansas State University, Manhattan, KS 66506 USA; Department of Mechanical and Nuclear Engineering, Kansas State University, Manhattan, KS 66506 USA; International Programs of the College of Agriculture and Life Science, Cornell University, Ithaca, NY 14853 USA; Wheat Genetics Resource Center, Department of Plant Pathology and Department of Agronomy, Kansas State University, Manhattan, KS 66506 USA

**Keywords:** Unmanned aerial vehicles/systems (UAV/UAS), Wheat, High-throughput phenotyping, Remote sensing, GNDVI, Plot extraction

## Abstract

**Background:**

Low cost unmanned aerial systems (UAS) have great potential for rapid proximal measurements of plants in agriculture. In the context of plant breeding and genetics, current approaches for phenotyping a large number of breeding lines under field conditions require substantial investments in time, cost, and labor. For field-based high-throughput phenotyping (HTP), UAS platforms can provide high-resolution measurements for small plot research, while enabling the rapid assessment of tens-of-thousands of field plots. The objective of this study was to complete a baseline assessment of the utility of UAS in assessment field trials as commonly implemented in wheat breeding programs. We developed a semi-automated image-processing pipeline to extract plot level data from UAS imagery. The image dataset was processed using a photogrammetric pipeline based on image orientation and radiometric calibration to produce orthomosaic images. We also examined the relationships between vegetation indices (VIs) extracted from high spatial resolution multispectral imagery collected with two different UAS systems (eBee Ag carrying MultiSpec 4C camera, and IRIS+ quadcopter carrying modified NIR Canon S100) and ground truth spectral data from hand-held spectroradiometer.

**Results:**

We found good correlation between the VIs obtained from UAS platforms and ground-truth measurements and observed high broad-sense heritability for VIs. We determined radiometric calibration methods developed for satellite imagery significantly improved the precision of VIs from the UAS. We observed VIs extracted from calibrated images of Canon S100 had a significantly higher correlation to the spectroradiometer (r = 0.76) than VIs from the MultiSpec 4C camera (r = 0.64). Their correlation to spectroradiometer readings was as high as or higher than repeated measurements with the spectroradiometer per se.

**Conclusion:**

The approaches described here for UAS imaging and extraction of proximal sensing data enable collection of HTP measurements on the scale and with the precision needed for powerful selection tools in plant breeding. Low-cost UAS platforms have great potential for use as a selection tool in plant breeding programs. In the scope of tools development, the pipeline developed in this study can be effectively employed for other UAS and also other crops planted in breeding nurseries.

## Background

In a world of finite resources, climate variability, and increasing populations, food security has become a critical challenge. The rates of genetic improvement are below what is needed to meet projected demand for staple crops such as wheat [1]. The grand challenge remains in connecting genetic variants to observed phenotypes followed by predicting phenotypes in new genetic combinations. Extraordinary advances over the last 5–10 years in sequencing and genotyping technology have driven down the cost and are providing an abundance of genomic data, but this only comprise half of the equation to understand the function of plant genomes and predicting plant phenotypes [2, 3]. High throughput phenotyping (HTP) platforms could provide the keys to connecting the genotype to phenotype by both increasing the capacity and precision and reducing the time to evaluate huge plant populations. To get to the point of predicting the real-world performance of plants, HTP platforms must innovate and advance to the level of quantitatively assessing millions of plant phenotypes. To contribute to this piece of the challenge, we describe here a semi-automated HTP analysis pipeline using a low cost unmanned aerial system (UAS) platform, which will increase the capacity of breeders to assess large numbers of lines in field trials.

A plant phenotype is a set of structural, morphological, physiological, and performance-related traits of a given genotype in a defined environment [4]. The phenotype results from the interactions between a plant’s genes and environmental (abiotic and biotic) factors. Plant phenotyping involves a wide range of plant measurements such as growth development, canopy architecture, physiology, disease and pest response and yield. In this context, HTP is an assessment of plant phenotypes on a scale and with a level of speed and precision not attainable with traditional methods [5], many of which include visual scoring and manual measurements. To be useful to breeding programs, HTP methods must be amenable to plot sizes, experimental designs and field conditions in these programs. This entails evaluating a large number of lines within a short time span, methods that are lower cost and less labor intensive than current techniques, and accurately assessing and making selections in large populations consisting of thousands to tens-of-thousands of plots. To rapidly characterize the growth responses of genetically different plants in the field and relate these responses to individual genes, use of information technologies such as proximal or remote sensing and efficient computational tools are necessary.

In recent years, there has been increased interest in ground-based and aerial HTP platforms, particularly for applications in breeding and germplasm evaluation activities [6–8]. Ground-based phenotyping platforms include modified vehicles deploying proximal sensing sensors [9–12]. Measurements made at a short distance with tractors and hand-held sensors that do not necessarily involve measurements of reflected radiation, are classified as proximal sensing. Proximal, or close-range sensing, is expected to provide higher resolution for phenotyping studies as well as allowing collection of data with multiple view-angles, illumination control and known distance from the plants to the sensors [13]. However, these ground-based platforms do have limitations mainly on the scale at which they can be used, limitations on portability and time required to make the measurements in different field locations.

As a complement to ground-based platforms, aerial-based phenotyping platforms enable the rapid characterization of many plots, overcoming one of the limitations associated with ground-based phenotyping platforms. There is a growing body of literature showing how these approaches in remote and proximal sensing enhance the precision and accuracy of automated high-throughput field based phenotyping techniques [14–16]. One of the emerging technologies in aerial based platforms is UAS, which have undergone a remarkable development in recent years and are now powerful sensor-bearing platforms for various agricultural and environmental applications [17–21]. UAS can cover an entire experiment in a very short time, giving a rapid assessment of all of the plots while minimizing the effect of environmental conditions that change rapidly such as wind speed, cloud cover, and solar radiation. UAS enables measuring with high spatial and temporal resolution capable of generating useful information for plant breeding programs.

Different types of imaging systems for remote sensing of crops are being used on UAS platforms. Some of the cameras used are RGB, multispectral, hyperspectral, thermal cameras, and low cost consumer grade cameras modified to capture near infrared (NIR) [2, 9, 21–23]. Consumer grade digital cameras are widely used as the sensor of choice due to their low cost, small size and weight, low power requirements, and their potential to store thousands of images. However, consumer grade cameras often have the challenge of not being radiometrically calibrated. In this study, we evaluated the possibility of using traditional radiometric calibration methods developed for satellite imagery to address this issue.

Radiometric calibration accounts for both variations from photos within an observation day along with changes between different dates of image. The result of radiometric calibration is a more generalized and, most importantly, repeatable, method for different image processing techniques (such as derivation of VIs, change detection, and crop growth mapping) applied to the orthomosaic image instead of each individual image in a dataset. There are well-established radiometric calibration approaches for satellite imagery. However, these approaches are not necessarily applicable in UAS workflows due to several factors such as conditions of data acquisition during the exact time of image capture using these platforms.

Empirical line method is a technique often applied to perform atmospheric correction to convert at-sensor radiance measurements to surface reflectance for satellite imagery. This technique can be modified to apply radiometric calibration and convert digital numbers (DNs) to reflectance values [24]. The method is based on the relationship between DNs and surface reflectance values measured from calibration targets located within the image using a field spectroradiometer. The extracted DNs from the imagery are then compared with the field-measured reflectance values to calculate a prediction equation that can be used to convert DNs to reflectance values for each band [25].

Advances in platform design, production, standardization of image geo-referencing, mosaicking, and information extraction workflow are needed to implement HTP with UAS as a routine tool in plant breeding and genetics. Additionally, developing efficient and easy-to-use pipelines to process HTP data and disseminate associated algorithms are necessary when dealing with big data. The primary objective of this research was to develop and validate a pipeline for processing data captured by consumer grade digital cameras using a low cost UAS to evaluate small plot field-based research typical of plant breeding programs.

## Methods

### Study area

**Fig. 1:**
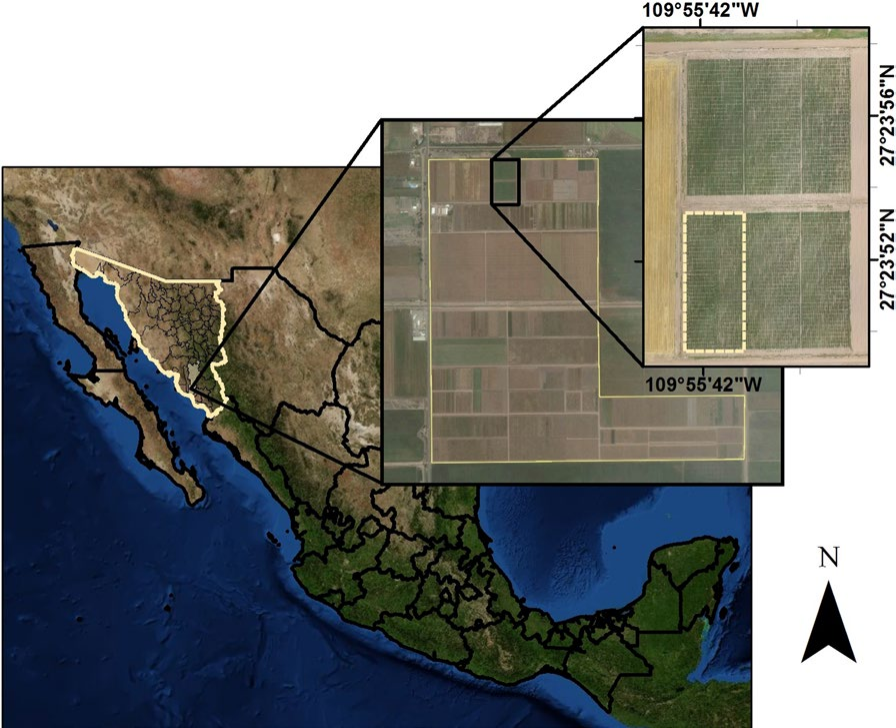
Location of the study area with late sown heat stress wheat trials in 2015 on the Norman E Borlaug Experiment Station at Ciudad Obregon, Sonora

The study was conducted at Norman E Borlaug Experiment Station at Ciudad Obregon, Sonora, Mexico (Fig. 1). The experiment consisted of 1092 advanced wheat lines distributed in 39 trials, each trial consisting of 30 entries. Each trial was arranged as an alpha lattice with three replications. The experimental units were 2.8 m × 1.6 m in size consisting of paired rows at 0.15 m spacing, planted on two raised beds spaced 0.8 m apart. The trials were planted later than optimal on February 24, 2015 to simulate heat stress with temperatures expected to be above optimum for the entire growing season (the local recommended planting date is November 15 to December 15). Irrigation and nutrient levels in the heat trials were maintained at optimal levels, as well as weed, insect and disease control. Grain yield was obtained by using a plot combine harvester.

### Platforms and cameras

We evaluated two unmanned aerial vehicles (UAV); the IRIS+ (3D Robotics, Inc, Berkeley, CA 94710, USA) and eBee Ag (senseFly Ltd., 1033 Cheseaux-Lausanne, Switzerland).

The IRIS+ is a low cost quadcopter UAV with a maximum payload of 400 g. The open-source Pixhawk autopilot system was programmed for autonomous navigation for the IRIS+ based on ground coordinates and the ‘survey’ option of Mission Planner (Pixhawk sponsored by 3D Robotics, www.planner.ardupilot.com). The IRIS+ is equipped with an uBlox GPS with integrated magnetometer. A Canon S100 modified by MaxMax (LDP LLC, Carlstadt, NJ 07072, USA, www.maxmax.com) to Blue–Green–NIR (400–760 nm) was mounted to the UAV with a gimbal designed by Kansas State University. The gimbal compensates for the UAV movement (pitch and roll) during the flight to allow for nadir image collection.

The eBee Ag, designed as a fixed wing UAV for application in precision agriculture has a payload of 150 g. This UAV was equipped with MultiSpec 4C camera developed by Airinov (Airinov, 75018 Paris, France, www.airinov.fr/en/uav-sensor/agrosensor/) and customized for the eBee Ag. It contains four distinct bands with no spectral overlap (530–810 nm): green, red, red-edge, and near infrared bands, and is controlled by the eBee Ag autopilot during the flight.

For ‘ground-truth’ validation, spectral measurements were taken using ASD VNIR handheld point-based spectroradiometer (ASD Inc., Boulder, CO, http://www.asdi.com/) with a wavelength range of 325–1075 nm, a wavelength accuracy of ±1 nm and a spectral resolution of <3 nm at 700 nm with a fiber optics of 25° (aperture) full conical angle. The instrument digitizes spectral values to 16 bits. Table 1 summarizes the specification for the cameras and sensor used in this study.

**Table 1:** Sensor specification

Sensor	Platform (UAV)	Sensor resolution (MP)	Focal length (mm)	Full width at half maximum (FWHM)	Peak wavelength
Canon S100	IRIS+	12.1	5.2	Blue: 400–495 Green: 490–550 NIR: 680–760	Blue: 460 Green: 525 NIR: 710
MultiSpec 4C	eBee Ag	1.2 (four sensors)	3.6	Green: 530–570 Red: 640–680 Rededge: 730–740 NIR: 770–810	Green: 550 Red: 660 Rededge: 735 NIR: 790

	Spectral resolution	Wavelength accuracy	Wavelength range
ASD spectroradiometer	<3 nm at 700 nm	±1 nm	325–1075 nm

### Ground control points

In order to geo-reference the aerial images, at the beginning of the season ten ground control points (GCPs) were distributed across the field. The GCPs were 25 cm × 25 cm square white metal sheets mounted on a 50 cm post. The GCPs were white to provide easy identification for the image processing software. The GCP coordinates were measured with a Trimble R4 RTK GPS, with a horizontal accuracy of 0.025 m and a vertical accuracy of 0.035 m. These targets were maintained throughout the crop season to enable more accurate geo-referencing of UAS aerial imagery and overlay of measurements from multiple dates.

### Reflectance calibration panel

For radiometric calibration, spectra of easily recognizable objects (e.g. gray scale calibration board) are needed. A black–gray–white grayscale board with known reflectance values was built and placed in the field during flights for further image calibration. This grayscale calibration panel met the requirements for further radiometric calibration including (1) the panel was spectrally homogenous, (2) it was near Lambertian and horizontal, (3) it covered an area many times larger than the pixel size of the Canon S100, and (4) covered a range of reflectance values [25].

The calibration panel used in this study had 6 levels of gray from 0 % being white and 100 % being black, printed on matte vinyl. This made it possible to choose several dark and bright regions in the images to provide a more accurate regression for further radiometric calibration analysis. Photos of the calibration panel were taken in the field before a UAS flight using the same Canon S100 NIR camera mounted on the IRIS+. Using an ASD spectroradiometer on a sunny day, the spectral reflectance was measured of the panel at a fixed altitude of 0.50 m from different angles.

### Field data collection

At the study site, image time series were acquired using IRIS+ and Canon S100 camera during the growing season as evenly spaced as possible, depending on the weather condition; April 10, April 22, April 28, May 6, and May 18, 2015.

Field data measurements for the experiment in this study included (1) IRIS+ carrying modified NIR Canon S100, and (2) eBee Ag carrying MultiSpec 4C camera, and (3) spectral reflectance measurements using handheld ASD spectroradiometer collected on May 6, 2015 when the trials were at mid grain filling. We took all field measurements within 1 h around solar noon to minimize variation in illumination and solar zenith angle [26]. This limited the number of plots we could measure using handheld spectroradiometer to 280 plots. Four spectra per plot were taken with the beam of the fiber optics placed at 0.50 m over the top of the canopy with a sample area of 0.04 m^2^. Due to this very small sampling area, great care was taken in data collection to make sure the measured spectral responses were only from the crops.

Both the eBee and IRIS+ were able to image a much larger area in the same time frame. Each system was flown using a generated autopilot path that covered the 2 ha experiment area with an average of 75 % overlap between images (Table 2).

**Table 2:** Flight information using IRIS**+** (multirotor) and eBee Ag (fixed wing) UASs, on May 6, 2015, at CIMMYT, Cd Obregon, Mexico

Camera	Platform (UAS)	Flight speed (m/s)	Altitude (m)	Percent overlap		No. of images	Image format	Spatial resolution (cm)
				Side (%)	Forward (%)			
Modified Canon S100	IRIS+	2	30	75	75	144	RAW (16 bits TIFF)^a^	0.8
MultiSPEC 4C	eBee Ag	10	48	75	75	40	10 bits TIFF	5

^a^RAW images of Canon S100 were converted to 16 bits TIFF imagery after pre-processing in Canon Digital Photo Professional software (DPP)

For the IRIS+ with Canon S100, all images were taken in RAW format (.CR2) to avoid loss of image information. The Canon S100 settings were TV mode, which allowed setting of a constant shutter speed. The aperture was set to be auto-controlled by the camera to maintain a good exposure level, and ISO speed was set to Auto with a maximum value of 400 to minimize noise in the images. Focus range was manually set to infinity. We used CHDK (www.chdk.wkia.com) to automate Canon S100’s functionality by running intervalometer scripts off an SD card in order to take pictures automatically at intervals during flight. The CHDK script allowed the UAS autopilot system to send electronic pulses to trigger the camera shutter. The camera trigger was set at the corresponding distance interval during the mission planning for the desired image overlap.

The eBee MultiSpec 4C camera had a predefined setting by Sensefly; ISO and shutter speed was set to automatic, maximum aperture was set to f/1.8 and focal distance was fixed at 4 mm.

### Developing an image processing pipeline for HTP

**Fig. 2:**
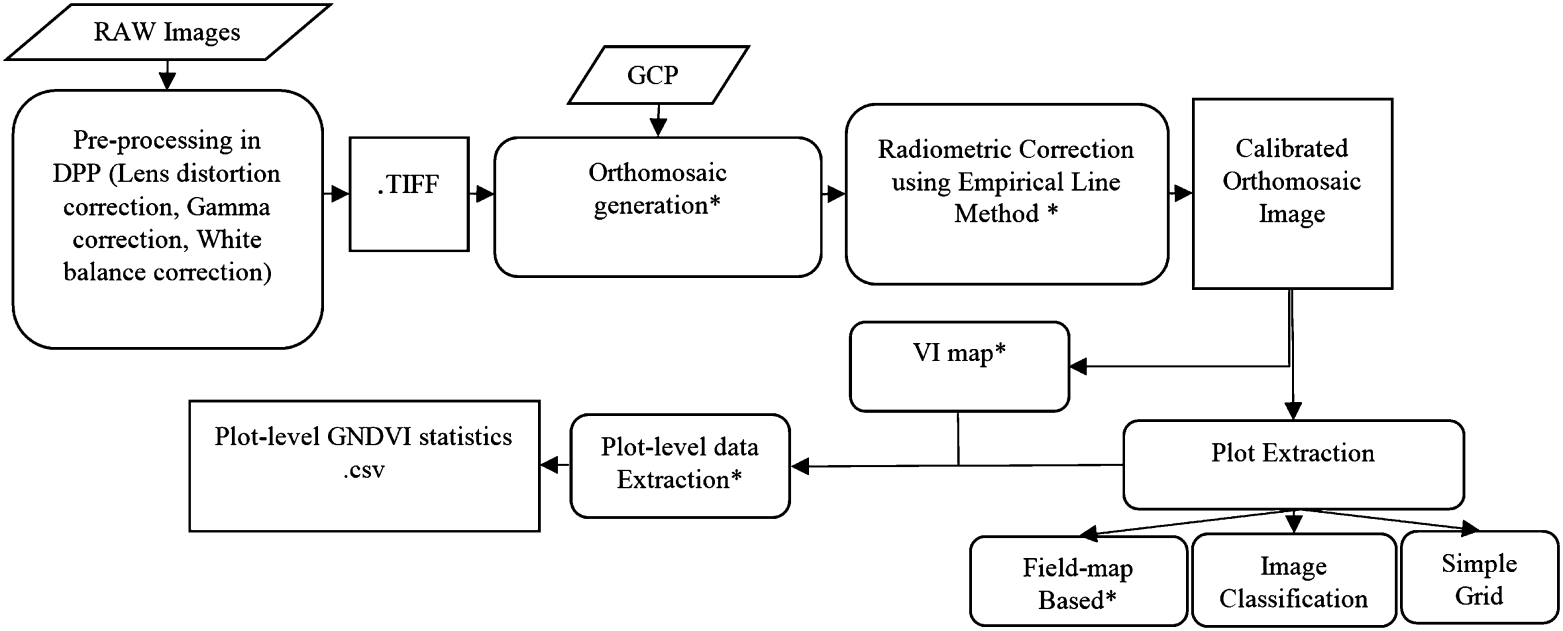
Image processing workflow for the low cost UAS imaging system. The developed pipeline steps are as follows: image pre-processing using DPP software, Orthomosaic Generation using Python scripting in PhotoScan, image radiometric correction using empirical line method commonly used for satellite imagery, extraction of wheat plot boundary, and calculation of different VI’s. *Asterisk* designates steps done using Python script

In this research, a UAS-based semi-automated data analyses pipeline was developed to enable HTP analysis of large breeding nurseries. Data analysis was primarily conducted using Python scripts [27, 28]. The dataset used to develop the image processing pipeline was collected on May 6, 2015 with the IRIS+ flights carrying the modified NIR Canon S100 camera. In order to analyze hundreds of images taken by a UAS that represent the entire experiment area, we developed a semi-automated data analysis pipeline. The developed pipeline completed the following steps: (1) image pre-processing, (2) georeferencing and orthomosaic generation, (3) image radiometric calibration, (4) calculation of different VIs and (5) plot-level data extraction from the VI maps. The overall workflow of the developed pipeline is presented in Fig. 2, and a detailed description of each segment is provided.

The RAW images of Canon S100 (.CR2 format) were pre-processed using Digital Photo Professional (DPP) software developed by Canon (http://www.canon.co.uk/support/camera_software/). This software to convert RAW to Tiff also included lens distortion correction, chromatic aberration, and gamma correction. For white balance adjustment, we used the pictures of grayscale calibration panel taken during the flights. After pre-processing the images were exported as a tri-band 16 bit linear TIFF image.

#### Image stitching and orthomosaic generation

We then generated a geo-referenced orthomosaic image using the TIFF images. There are several software packages for mosaicking UAS aerial imagery, all based around the scale-invariant feature transform algorithm (SIFT) algorithm for feature matching between images [29]. While there are slight differences between packages each performs the same operation namely loading photos; aligning photos; importing GCP positions; building dense point cloud; building digital elevation model (DEM); and finally generating the orthomosaic image. We used the commercial Agisoft PhotoScan package (Agisoft LLC, St. Petersburg, Russia, 191144) to automate the orthomosaic generation within a custom Python script. The procedure of generating the orthomosaic using PhotoScan comprises five main stages (1) camera alignment, (2) importing GCPs and geo-referencing, (3) building dense point cloud, (4) building DEM and (5) generating orthomosaic [30].

For alignment, PhotoScan finds the camera position and orientation for each photo and builds a sparse point cloud model; the software uses the SIFT algorithm to find matching points of detected features across the photos. PhotoScan offers two options for pair selection: generic and reference. In generic pre-selection mode the overlapping pairs of photos are selected by matching photos using the lower accuracy setting. On the other hand, in reference mode the overlapping pairs of photos are selected based on the measured camera locations. We examined both methods to generate the orthomosaic and compared the results.

With aerial photogrammetry the location information delivered with cameras is inadequate and the data does not align properly with other geo-referenced data. In order to do further spatial and temporal analysis with aerial imagery, we performed geo-referencing using GCPs. We used ten GCPs evenly distributed around the orthomosaic image. Geo-referencing with GCPs is the step that still requires manual input.

The main source of error in georeferencing is performing the linear transformation matrix on the model. In order to remove the possible non-linear deformation components of the model, and minimizing the sum of re-projection error and reference coordinate misalignment, PhotoScan offers an optimization of the estimated point cloud and camera parameters based on the known reference coordinates [30].

After alignment optimization, the next step is generating dense point clouds. Based on the estimated camera positions PhotoScan calculates depth information for each camera to be combined into a single dense point cloud [30]. PhotoScan allows creating a raster DEM file from a dense point cloud, a sparse point cloud or a mesh to represent the model surface as a regular grid of height values. Although most accurate results are calculated based on dense point cloud data.

The final step is orthomosaic generation. An orthomosaic can be created based on either mesh or DEM data. Mesh surface type can be chosen for models that are not referenced. The blending mode has two options (mosaic and average) to select how pixel values from different photos will be combined in the final texture layer. Average blending computes the average brightness values from all overlapping images to help reduce the effect of the bidirectional reflectance distribution function that is strong within low altitude wide-angle photography [31]. Blending pixel values using mosaic mode does not mix image details of overlapping photos, but uses only images where the pixel in question is located within the shortest distance from the image center. We compared these two methods by creating orthomosaic images using both mosaic and average mode.

#### Radiometric calibration

**Fig. 3:**
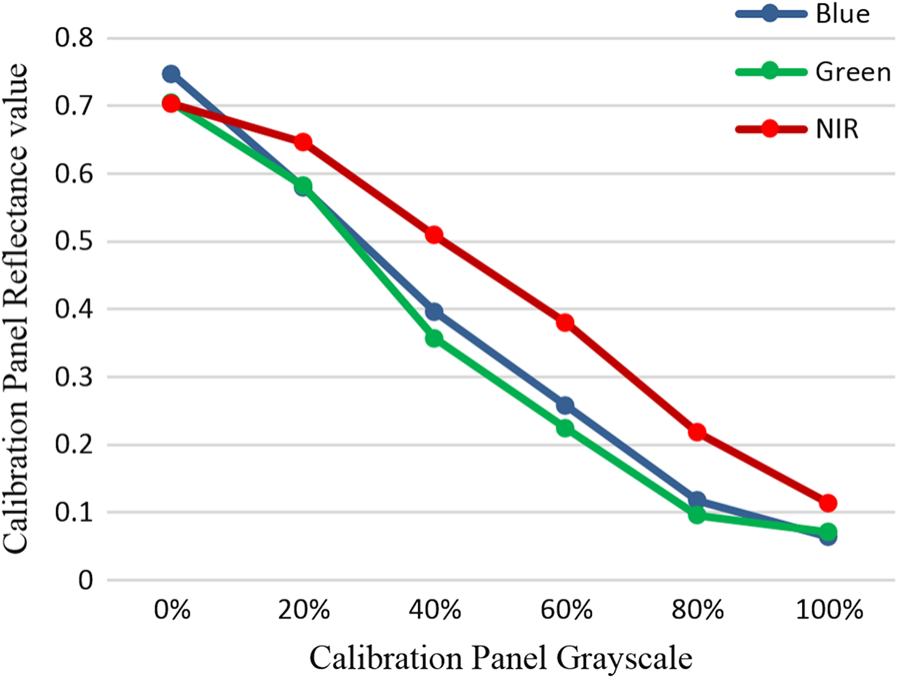
Empirical observation of non-linear relationship between percent gray values of the calibration panel and their corresponding mean reflectance values at the spectral region of Canon S100 wavebands (B: 400–495, G: 490–550, NIR: 680–760)

After the orthomosaic was completed, we then performed radiometric calibration to improve the accuracy of surface spectral reflectance obtained using digital cameras. We applied empirical line correction to each orthomosaic within the data set using field measurements taken with the ASD. The relationship between image raw DNs and their corresponding reflectance values is not completely linear for all the bands for Canon S100 camera and most digital cameras (Fig. 3). To relate remote sensing data to field-based measurements we used a modified empirical line method [32]. We used the grayscale calibration board combined with reflectance measurements from the ASD spectroradiometer to find the relationship between DNs extracted form Canon S100 and surface reflectance values measured from grayscale calibration board using a field spectroradiometer. We then calculated the prediction equations for each band separately to convert DNs to reflectance values.

We calculated the mean pixel value of the calibration panel extracted from images for each band separately, and plotted against the mean band equivalent reflectance (BER) of field spectra [33, 34]. We found a linear relation between the natural logarithm of image raw DN values and their corresponding BER, although the relation between image raw DNs and their corresponding BER was not completely linear for all bands of Canon S100 (Fig. 3). We derived the correction coefficient needed to fit uncalibrated UAS multispectral imagery to field-measured reflectance spectra. Using the correction coefficient for each band, we then performed this correction on the entire image.

With the corrected orthomosaic, a map showing the vegetation indices can be generated. The most used VIs derivable from a three-band multispectral sensor are: normalized difference vegetation index (NDVI), and green normalized difference vegetation index (GNDVI) (Table 4). NDVI is calculated from reflectance measurements in the red and NIR portion of the spectrum and its values range from −1.0 to 1.0. GNDVI is computed similarly to the NDVI, where the green band is used instead of the red band and its value ranges from 0 to 1.0. Like NDVI, this index is also related to the proportion of photosynthetically absorbed radiation and is linearly correlated with leaf area index (LAI) and biomass [22, 35, 36].

#### Field plot extraction

In order to get useful information about each wheat plot in the field, we need to extract plot level data from the orthomosaic VI image. Individual wheat plot boundaries need to be extracted and defined separately from images with an assigned plot ID that defines their genomic type. We examined three approaches for extracting plot boundaries from the orthomosaic image: a simple grid based plot extraction, field-map based plot extraction, and image classification/segmentation.

To apply a simple grid superimposed on top of the orthomosaic image we used the Fishnet function using Arcpy scripting in ArcGIS. This function creates a net of adjacent rectangular cells. The output is polygon features defining plot boundaries. Creating a fishnet requires two basic pieces of information: the spatial extent of the desired net, and the number of rows and columns. The number of rows and ranges (columns) and height and width of each cell in the fishnet can be defined by the user. The user can also define the extent of the grid by supplying both the minimum and maximum x- and y-coordinates.

The next method we evaluated in this study is a field-map based method. Experimental or field data are usually stored in a spreadsheet. To accomplish this, we reformatted the field map in order to represent the plots in the field; the first row of the excel sheet is the length (or width, depending on how plots are located in the field) of the plots, and the first column is the width (or length). Each cell in the excel sheet is the plot ID and other information regarding that particular plot available in the field map.

In this approach, first we created a KML file from the field map (.CSV) using Python [27], we generated polygon shapefiles with known size for each plot from KML file, and assigned plot ID to each plot using the field map. To define the geographic extent of the field, the python script has inputs for the coordinate of two points in the field: the start point of the first plot on the top right and the end point of the last plot on the bottom left. The script starts from the top right and builds the first polygon using the defined plot size, and skips the gap between plots and generates the next one until it gets to the last plot on the bottom left. In this approach the plot IDs are assigned automatically and simultaneously from the field map excel sheet. The most important advantage of this approach is that it can be generalized to other crop types as long as the field map is provided and the plots are planted in regular distance and have a consistent size within a trial.

The third method is to extract wheat plot boundaries from the orthomosaic image directly. To examine this method, we used the April 10 data set instead of May 6. The reason for choosing this set of data was that in this dataset we had the most distinguished features (e.g. vegetation vs. bare soil) in the image compared to the image data taken on May 6. We classified the GNDVI image created from the orthomosaic image into four classes: “Wheat”, “Shadow”, “Soil”, and a class named “Others” for all the GCP targets in the field. The class “shadow” stands for the shadow projected on the soil surface. In this supervised classification, we defined training samples and signature files for each defined class. After classifying the image, we merged the “Shadow”, “Soil” and “Others” classes together and ended up with two classes: “Wheat”, and “Non-wheat”. The classified image often needs further processing to clean up the random noise and small isolated regions to improve the quality of the output. With a simple segmentation, we extracted wheat plot polygons from the image.

This method works best with high-resolution imagery taken at the beginning of the growing season when all the classes are clearly distinguishable from each other on the image. Otherwise time consuming post-processing is needed in order to separate the mixed classes.

To analyze the result of VI extraction from the orthomosaic, we used the zonal statistics plugin in the open source software QGIS. We calculated several values of the pixels of orthomosaic (such as the average VI values, Min, Max, standard deviation, majority, minority and also the median of VI values for each plot and the total count of the pixels that are within a plot boundary), using the polygonal vector layer of plot boundaries generated by one of the methods above. We then merged the data from the field map spreadsheet (plot ID, entry numbers, trial name, number of rep, number of block, row and column, and other information on planting) with the generated plots statistics.

### Accuracy evaluation of different types of aerial imagery sensors and platforms

To evaluate the accuracy and reliability of our low cost UAS and consumer grade camera, we calculated correlations between calibrated DN values from the orthomosaic images of Canon S100 and MultiSpec 4C camera and band equivalent reflectance (BER) of field spectra. For ground-truth evaluation we used the ASD Fieldspectro 2 spectroradiometer to measure spectral reference of 280 sample plots after running the UAS within the time window of 1 h around noon. The ASD spectroradiometer measures a wavelength range of 325–1075 nm, a wavelength accuracy of ±1 nm and a spectral resolution of <3 nm at 700 nm (Table 1). Using the ASD, the top of canopy reflectance was measured approximately 0.5 m above the plants. To account for the very small field of view of this sensor, we constantly monitored the spectral response curves to avoid measuring mixed pixels in our samplings and we averaged four reflectance readings within each sample plots. Reflectance measurements were calibrated at every 15 plots using a white (99 % reflectance) Spectralon calibration panel.

To evaluate the accuracy of each platform, we calculated the correlations between different VIs extracted from Canon S100 and VIs extracted from MultiSpec 4C compared to the BER of field spectra. We calculated BER for each camera separately by taking the average of all the spectroradiometer bands that are within the full width at half maximum (FWHM) of each instrument (Table 1). The Pearson correlation between the average VI values of the sample plots and field spectra for each platform was calculated. The resulting correlation coefficients were tested using a two-tail test of significance at the p ≤ 0.05 level. Accuracy assessment was based on computing the root mean square error (RMSE) for GNDVI image by comparing the pixel values of the reflectance image to the corresponding BER of field spectra at the sample sites not selected to generate the regression equations.

### Broad-sense heritability

Broad-sense heritability, commonly known as repeatability, is an index used by plant breeders and geneticists to measure precision of a trial [37–39]. The ratio of the genetic variance to the total phenotypic variance is broad-sense heritability (*H*^2^), which varies between 0 and 1. A heritability of 0 indicates there is no genetic variation contributing to phenotypic variation, and *H*^2^ of 1 if the entire phenotypic variation is due to genetic variation and no environmental noise. For the purpose of comparing phenotyping tools and approaches, broad-sense heritability calculated for measurements on a given set of plots under field conditions at the same point in time, is a direct assessment of measurement error as the genetics and environmental conditions are constant.

In order to assess the accuracy of the different HTP platforms, we calculated broad-sense heritability on an entry-mean basis for each individual trial and phenotypic sampling date on May 6, 2015. To calculate heritability we performed a two steps process where we first calculated the plot average and then fit a mixed model for replication, block and entry as random effects. Heritability was calculated from Eq. 1 [40].

1 $$H^{2} = \frac{{\sigma_{genotypic}^{2} }}{{\sigma_{phenotypic}^{2} }} = \frac{{\sigma_{genotypic}^{2} }}{{\sigma_{genotypic}^{2} + \frac{{\sigma_{error}^{2} }}{replication}}}$$

where $\sigma_{genotypic}^{2}$ is genotypic variance and $\sigma_{error}^{2}$ is error variance.

## Results and discussion

### Developing an image processing pipeline for HTP

To evaluate the potential of UAS for plant breeding programs, we deployed a low cost, consumer grade UAS and imaging system at multiple times throughout the growing season. Using the aerial imagery collected on May 6, we built a robust pipeline scripted in Python that facilitated image analysis and allowed a semi-automated image analysis algorithm to run with minimum user support. The only requirement for manual input in the processing pipeline is to validate the GCP positions. This pipeline was tested on eight temporal datasets collected from IRIS+ flights over heat trials during the growing season.

The individual parameters selected during orthomosaic generation and plot extraction can have a large impact on the quality of the orthomosaic and subsequent data analysis. Because of the multiple parameter settings that users could choose, we investigated how changing various parameters would affect the data quality. To obtain more accurate camera position estimates, we selected higher accuracy at the alignment step. For pair selection mode, we chose reference since in this mode the overlapping pairs of photos are selected based on the estimated camera locations (i.e. from the GPS log of the control system). For the upper limit of feature points on every image to be taken into account during alignment stage, we accepted the default value of 40,000. We imported positions for all of the ten GCPs available on the field to geo-reference the aerial images. The GCPs were also used in the self-calibrating bundle adjustment to ensure correct geo-location and to avoid any systematic error or block deformation. To improve georeferencing accuracy, we performed alignment optimization.

**Fig. 4:**
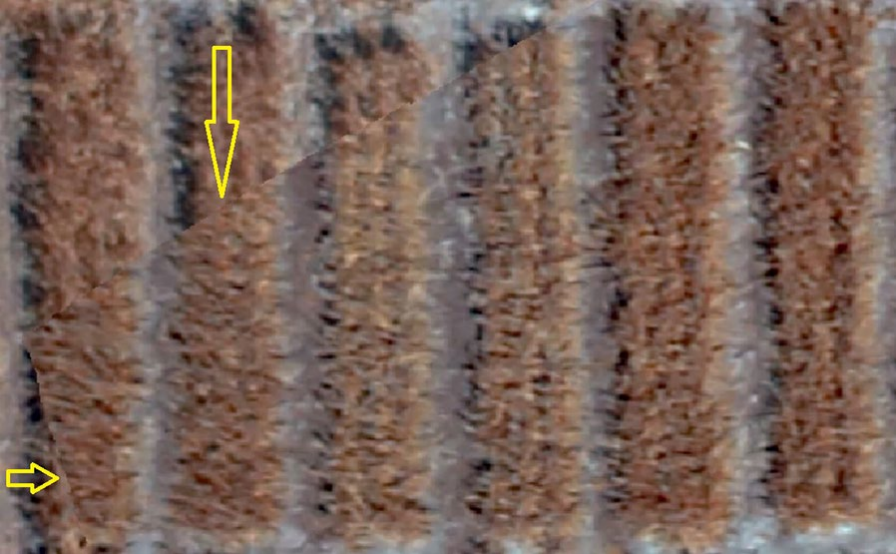
An example of artifact in the orthomosaic generated by Mosaic blending method from IRIS+ aerial imagery. The *yellow arrows* point to two locations in the orthomosaic where artifacts from merging different original images are evident

Finally, to generate orthomosaic, we tested the two methods of blending: mosaic and average. We found the orthomosaic generated by the mosaic method gives more quality for the orthomosaic and texture atlas than the average mode. On the other hand, mosaic blending mode generated artifacts in the image since it uses fewer images by selecting pixels with the shortest distance from the image center (Fig. 4). We examined the correlation between orthomosaic generated from these two approaches and the ground truth data and found the averaging method has slightly higher correlation of 0.68 compared to mosaic method with the correlation of 0.65.

The 16 bit orthomosaic TIFF image was generated semi-automatically using python scripting in PhotoScan software with a resolution of 0.8 cm, matching the ground sampling distance (GSD) of the camera. Due to the short flight times (less than 20 min) and low flying altitude (30 meters above ground level), we assume there is no need for atmospheric corrections [28].

To convert DNs to reflectance values, a modified empirical approach was implemented using pixel values of IRIS+ aerial imagery and field-based reflectance measurements from handheld spectroradiometer. Calibration Eqs. (2, 3, and 4) calculated from non-linear relationship between average field spectra and relative spectra response of each band separately were determined as:

2 $$\begin{aligned} & Reflectance_{Blue} = - \exp ( - \log (4.495970e^{ - 01} + (3.191189e^{ - 05} ) * DN_{Blue} )) \\ & (r^{2} = 0.98 ,\;P < 0.001) \\ \end{aligned}$$

3 $$\begin{aligned} & Reflectance_{Green} = - \exp ( - \log (4.705485e^{ - 01} + (3.735676e^{ - 05} ) * DN_{Green} )) \\ & (r^{2} = 0.97, \, P < 0.001) \\ \end{aligned}$$

4 $$\begin{aligned} & Reflectance_{NIR} = - \exp ( - \log (1.197440e^{ + 00} + (4.712299e^{ - 05} ) * DN_{NIR} )) \\ & (r^{2} = 0.94, \, P < 0.001) \\ \end{aligned}$$

We applied each calibration equation to their corresponding band image and converted the image raw DNs into reflectance values.

### Wheat plot boundary extraction results

We evaluated multiple approaches for defining and overlaying plot coordinate boundaries for the large field trials. As breeding trials are regularly containing thousands or tens of thousands of plots, an automated or semi-automated approach to generating and overlaying plot boundaries is needed.

#### Simple-grid based method

**Fig. 5:**
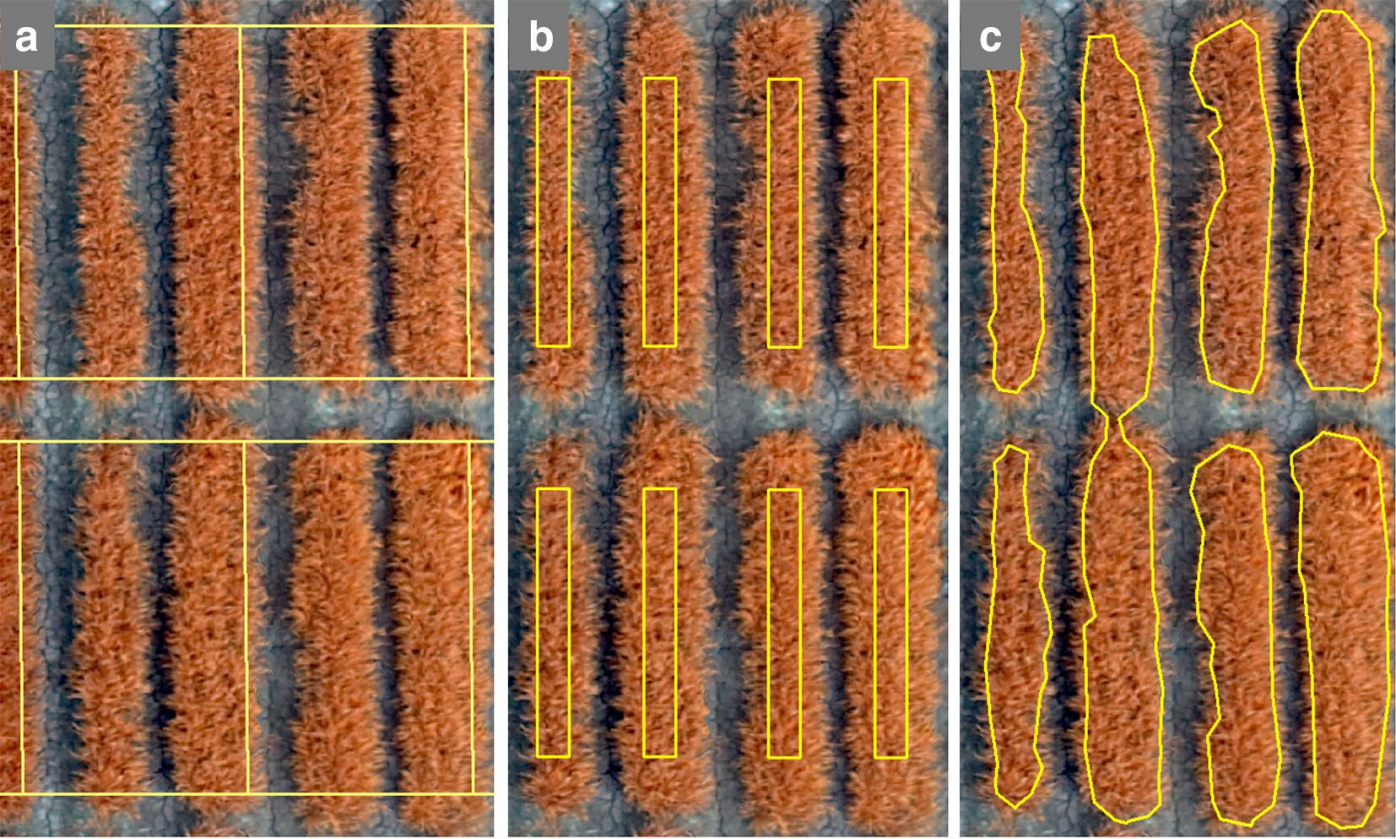
Results of different plot extraction method overlay on a subset of plots from IRIS+ calibrated orthomosaic. **a** Simple-grid plots, **b** field-map based method, **c** image classification approach and an example of misclassification; this example confirms the need of post processing for image classification method

We first implemented a simple overlay grid (Fig. 5a) using QGIS open source software. This method, although fast and easy, is not accurate for plot assigning as it does not account for within plot gaps (for plots planted in beds), gaps between plots and also gaps between each range of wheat plots. It simply generates polygons attached to each other and not spaced as needed to correctly capture the actual plot boundaries (Fig. 5a). In the example below, it can also be noticed that a boundary polygon may cover part of the neighbor wheat plot, which is due to the assumption of a fixed plot boundary size in this method. To account for the gap between plots, beds and also between ranges of plots, we developed a field map based method.

#### Field-map based method

To accurately reflect the actual field planting and plot size, we developed a script to overlay defined plot sizes with known spacing. We reformatted the field map using the information in the spreadsheet such as: plot ID, and plot’s location based on the block number, column and row’s number, and also knowing the plots were planted on beds with a plot size of 2.8 m length and 1.6 m wide. This method allows us to eliminate border effect by changing the plot size to 2.3 m by 1.5 m. In the reformatted field map spreadsheet, the first row of the excel sheet is the length of the plots (1.5 m) and the length of the gap between plots in beds (0.15 m) and gaps between each bed (0.8 m). The first column is the width of wheat plots (2.3 m) and the width of the gaps between each range (0.5 m). The cells in the reformatted spreadsheet are filled with the plot’s information including plot ID, trial name, and planting date. From this plot level information, we overlay plots of the defined size with plot-to-plot spacing that accurately reflects the field configuration (Fig. 5b).

#### Image classification plot extraction

To examine an image classification based plot extraction method, we first generated GNDVI from the orthomosaic image generated from the April 10 aerial imagery rather than the May 6 dataset analyzed in the rest of this study. The reason for choosing this set of data was that in this dataset from earlier in the season had the sharpest contrast between plots and the surrounding soil. We then applied a Maximum Likelihood Classification to the GNDVI image. We converted the classified image into polygons to obtain plot shape polygons (Fig. 5c). This approach is more reliable if the early season aerial imagery is provided. The drawback with this approach is that it requires post-processing and cleanup of the final result if the classes are not quite distinguished and separated from each other in the orthomosaic image. Frequently, this approach did not separate plots properly and manual editing was required (Fig. 5c). As it can be seen in the Fig. 5c, the two plots slightly merged with each other and it caused the classification to classify them as one single plot. To have more accurate results from image classification method, the presence of early season imagery is needed, since this process can have more post-processing and cleanup if the wheat plots and the gaps between plots are not visually discrete from each other in the images.

### Plot-level GNDVI extraction results

After generating VI maps, we compared these three plot-level extraction techniques using GNDVI values extracted form canon S100 aerial imagery and the corresponding values extracted from spectroradiometer in the field. The field-map based approach for plot polygons generated the best correlation to spectroradiometer measurements, while the classification technique had the lowest correlation with ground-truth data (Table 3). The lower reliability of classification technique compared to field-map based, could be due to the absence of early season imagery. In generating plot boundaries using field-map based, we also considered a buffer around the polygons to avoid any possible mixed plants or border effects for a plot which likely improved performance. We then correlated plot level GNDVI calculated from the spectral reflectance for those same plots using different boundary plot extraction methods (Table 3). From the comparison of different plot extraction methods with correlation to spectral reflectance we found that the boundaries defined using the map-based algorithm were superior to both the simple grid and the classification-based approach (Table 3).

**Table 3:** Correlation analysis between mean GNDVI values extracted from raw Canon S100 digital number values, Calibrated Canon S100 digital number values and corresponding band equivalent reflectance (BER) GNDVI values from the spectroradiometer using different plot boundary extraction method

	Raw digital numbers	Calibrated digital numbers		
	Field-map based	Field-map based	Classification	Simple-grid
Spectroradiometer BER	0.68	0.76	0.58	0.68

### Empirical line correction

Using Python scripting in QGIS, we generated different VI maps from the calibrated orthomosaic image and extracted plot boundaries using the field-map based method. Using the zonal statistics plugin, we then calculated statistic values of each plot of wheat in the orthomosaic. For each individual plot, the average VI value was used for further analysis and correlation. We tested the utility of applying an empirical line correction to improve the converting of digital numbers to reflectance values for the cameras. Using the same field-map based plot extraction, the correlation between raw GNDVI values extracted from the orthomosaic image and the BER increased from 0.68 to 0.76 after performing empirical line method as the radiometric calibration method.

### Comparison of different vegetation indices

To test the accuracy of VI from the digital cameras by comparison to the ASD spectroradiometer, we calculated BER for Canon S100 by taking the average of all the spectroradiometer bands that are within the FWHM of this camera (Table 1). For example, to calculate BER GNDVI for Canon S100, we averaged the reflectance values between 490 and 550 nm as green band, and averaged all the values between 680 and 760 nm as NIR band.

**Fig. 6:**
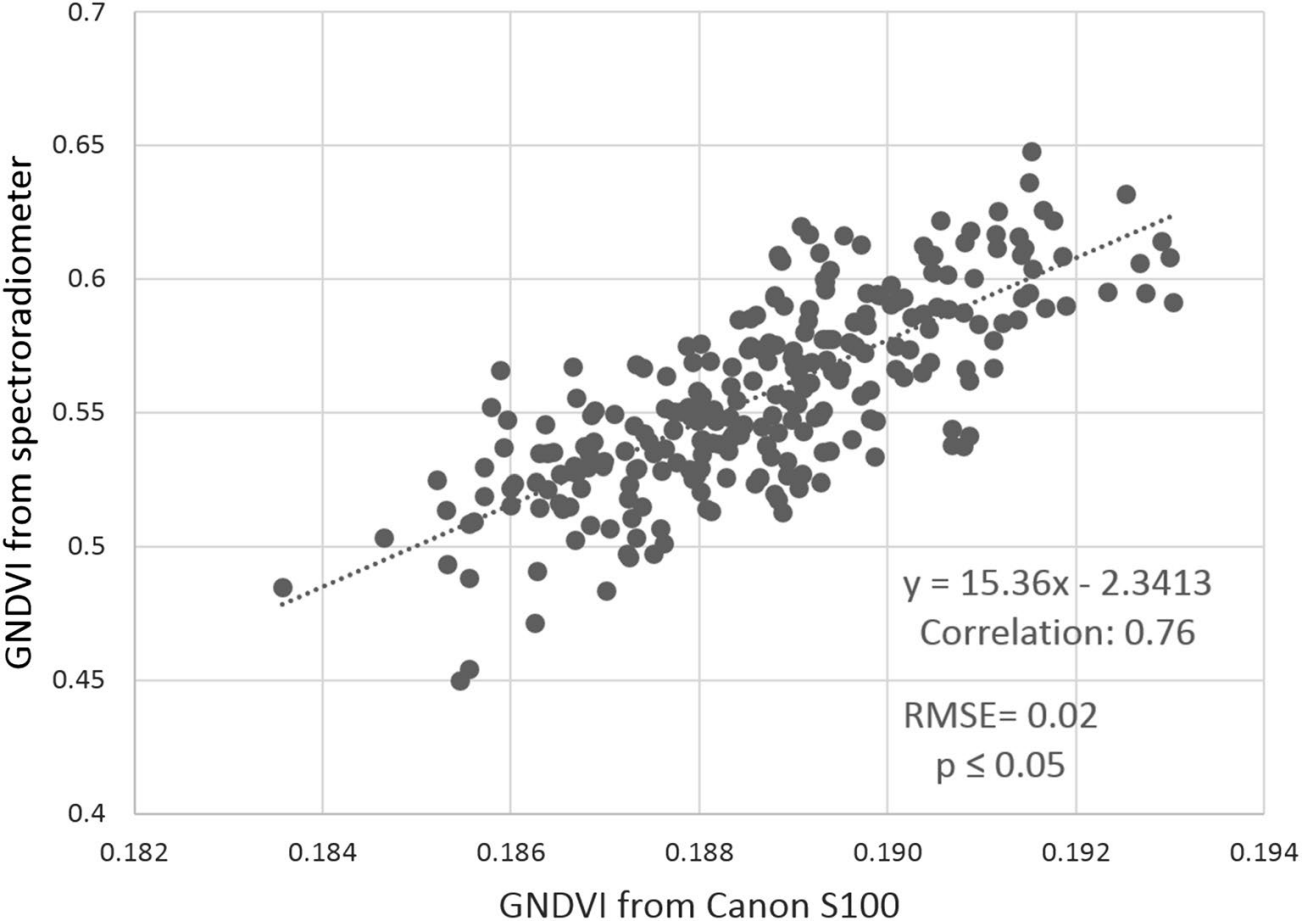
Linear relation between GNDVI from Canon S100 and BER GNDVI from spectroradiometer. Sample plot GNDVI values were extracted from calibrated orthomosaic of Canon S100 imagery, IRIS+ and band equivalent GNDVI for Canon S100 camera calculated from ASD spectroradiometer readings for 280 sample plots

**Fig. 7:**
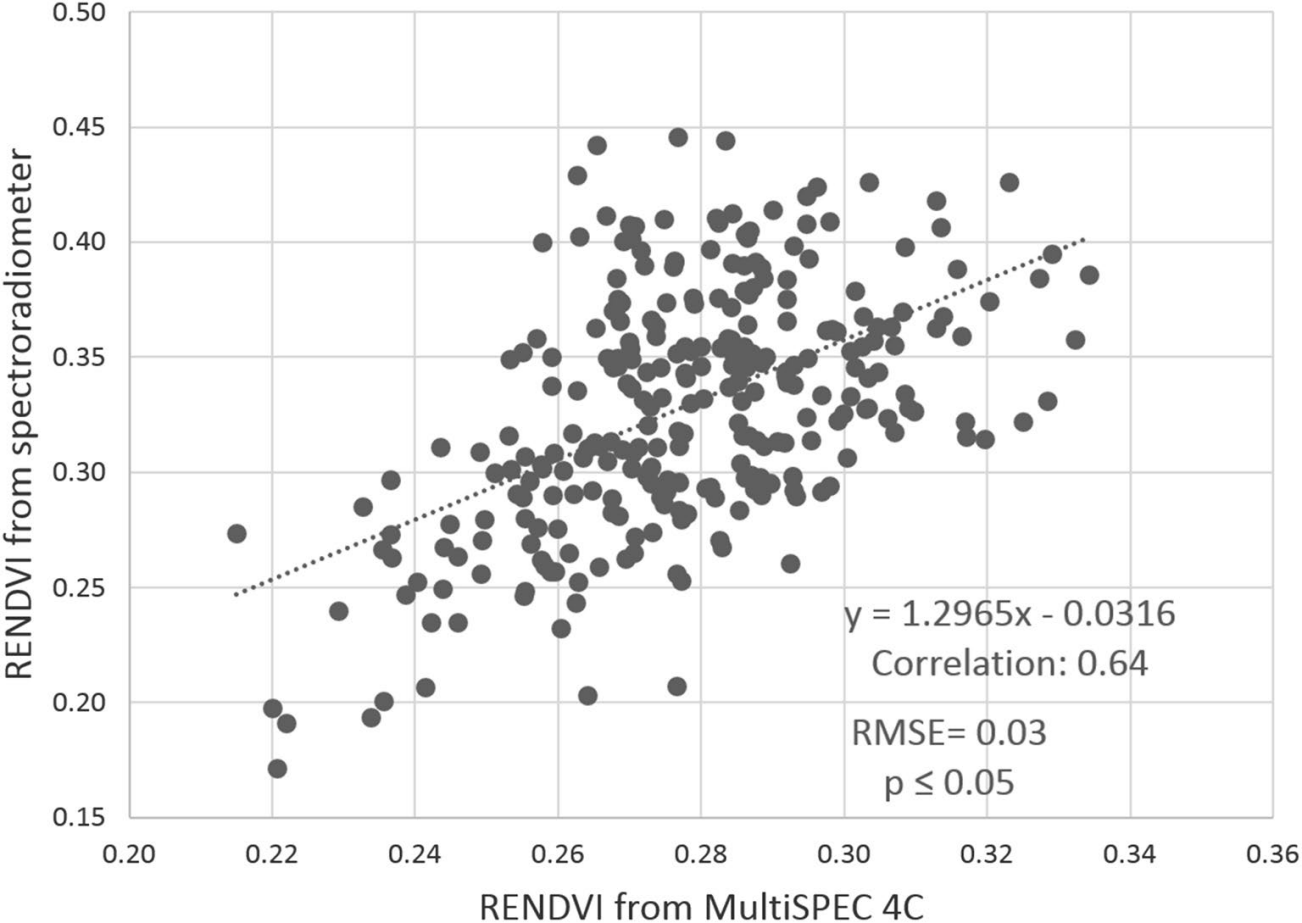
Scatterplot of the plot-level RENDVI from MultiSpec 4C imagery versus spectroradiometer. Plot-level RENDVI were extracted from orthomosaic generated from MultiSPEC 4C camera imagery using field-map based plot extraction method, and Band equivalent RENDVI for Spec 4C calculated from spectroradiometer reading for 280 sample plots

Among all the calculated VIs for the Canon S100, GNDVI had the highest correlation with the spectroradiometer (Table 4; Fig. 6). Since the MultiSpec 4C camera has 4 bands, NIR, red-edge, red and green, we were able to generate a red-edge normalized difference vegetation index (RENDVI) as well as other VIs. Using the developed pipeline, we extracted wheat plots from the generated orthomosaic for RENDVI by means of the field-map based method, which had also proved to be the best method of plot extraction for the MultiSpec 4C camera. The RENDVI plot values had higher correlation with spectroradiometer when compared to other VIs extracted from MultiSpec 4C camera (Fig. 7).

**Table 4:** Calculated vegetation indices for each cameras, based on their spectral bands and correlation between vegetation index values extracted from UAS imagery and band equivalent reflectance values from Spectroradiometer

Index	Formula	Correlation	
		Canon S100	MultiSpec 4C
NDVI	$\frac{NIR - Red}{NIR + Red}$	–	0.33
GNDVI	$\frac{NIR - Green}{NIR + Green}$	*0.68*	0.63
RENDVI	$\frac{NIR - Red\_edge}{NIR + Red\_edge}$	–	*0.64*
ENDVI	$\frac{NIR + Green - 2*Blue}{NIR + Green + 2*Blue}$	0.54	–
GIPVI	$\frac{NIR }{NIR + Green}$	0.59	–

The values from Canon S100 values are before applying modified empirical line correction method

GNDVI values for Canon S100, and RENDVI for MultiSpec 4C have higher correlation with spectroradiometer compoare to other vegetation indices (shown in italics font)

We found the correlation between VIs extracted from spectroradiometer and calibrated Canon S100 was significantly higher than the one with MultiSpec 4C camera. This could be due to lower flight altitude, and slower movements of multi-rotor IRIS+ compare to fixed wing eBee Ag, which results in higher resolution imagery captured by IRIS+/Canon S100. The MultiSpec 4C camera is a narrow band multispectral camera that does not have any spectral overlap in its band response. The better spectral performance could be negated by the lower resolution of the sensor, higher flight altitude, and faster travel speed of the fixed wing UAV. It was also clear that the modified empirical line correction also played a role in higher correlation of Canon S100 values with ground truth spectral measurements.

We also calculated the correlation between the spectra readings using the handheld spectroradiometer. As we had four readings per plot, we were able to determine the repeatability within the spectroradiometer measurements. We averaged the first two measurements of and last two measurements for each plot independently and calculated an overall correlation for 280 sample plots (Table 5). Based on this assessment, the UAS imaging had repeatability as good as, or better than the spectroradiometer.

**Table 5:** Correlation between repeated measurements from Handheld ASD for two different vegetation indices

	Correlation
Green normalized difference vegetation index; GNDVI (Canon S100)	0.62
Red-edge normalized difference vegetation index; RENDVI (MultiSpec 4C)	0.88

Four measurements were taken on each of 280 plots. To determine repeatability, the first two measurements were averaged and correlated to the average of the second two measurements on a per plot basis across all plots. The VIs presented are band equivalent indices for the Canon S100 (green normalized difference vegetation index; GNDVI) and MultiSpec 4C (red-edge normalized difference vegetation index: RENDVI) used in this study

### Broad sense heritability

As the Canon S100 and MultiSpec 4C measurements were taken at the same time on the same day and on the same plots, the environmental variance and genetic variance can be considered as negligible. Therefore, the broad sense heritability values (*H*^2^) can be interpreted as a level of precision of sensor measurement, which is the only remaining significant source of error variance. Overall the heritability for VIs was high. We found VIs derived from the Canon S100 imagery had a higher heritability than the MultiSpec 4C data for nine out of eleven trials (Table 6). The trials had lower, but still high, heritability for grain yield. There were some unexpected rains during the crop cycle followed by irrigation issues in the borders of the field, however, the heritability for yield was still above 0.60 which is a moderate repeatability for breading trials under heat (Table 6).

**Table 6:** Broad-sense heritability in 11 trials for vegetation indices and grain yield

Trial name	Broad-sense heritability (*H* ^2^)		
	Vegetation indices		Grain yield
	Canon S100 (GNDVI)	MultiSpec 4C (RENDVI)	
EYTBWBLHT_01	*0.89*	0.84	0.66
EYTBWBLHT_02	0.90	0.90	0.91
EYTBWBLHT_03	0.84	*0.90*	0.78
EYTBWBLHT_04	*0.87*	0.70	0.75
EYTBWBLHT_05	*0.81*	0.80	0.82
EYTBWBLHT_06	*0.73*	0.66	0.80
EYTBWBLHT_07	*0.83*	0.70	0.85
EYTBWBLHT_08	*0.64*	0.61	0.86
EYTBWBLHT_09	*0.78*	0.64	0.87
EYTBWBLHT_10	*0.66*	0.57	0.65
EYTBWBLHT_11	*0.72*	0.57	0.74

The VIs derived from the Canon S100 had a higher heritability than the MultiSpec 4C for nine out of eleven trials (shown in italics font)

## Conclusion

We developed a semi-automated pipeline for data analysis of a low cost UAS imagery. The raw images of a consumer grade digital camera were pre-processed and used as the input of the image-processing pipeline. During the mosaicking step, we found that the averaging method had marginally higher correlation of 0.68 compared to mosaic method with the correlation of 0.65. Using a modified empirical line method, we radiometrically calibrated the DN values extracted from canon S100 and compared the calibrated digital values with the reflectance values of the spectroradiometer to evaluate the comparability of our data. Our results confirm that radiometric calibration is important for consumer grade cameras to convert the DN values to reflectance measurements and can improve the value of the image data.

We examined three different ways of wheat plot extraction: simple grid, field-map based, and image classification. We found that the field-map based technique is more accurate and fastest compared to other techniques, and had a higher correlation to ground truth data. The advantage of this method is that it is applicable to any crop types as long as the field map is provided, and also this method is fully automated in Python.

We evaluated two types of UAS; (1) low cost: IRIS+ and (2) commercial grade agriculture-use: eBee Ag, each carrying different sensors with (1) low cost consumer grade camera: modified NIR Canon S100 on the IRIS+ and (2) a more specialized multispectral camera: MultiSpec 4C camera on the eBee. We compared their performance with ground-truth reflectance data and found overall good performance to spectroradiometer measurements. Based on correlation to the spectral readings and assessment of heritability, the Canon S100 had better performance than the MultiSpec 4C mounted on the fixed wing, which was likely a result of higher resolution of the sensor, lower altitude and slower travel speed for the Canon S100 carried on the quadcopter IRIS+.

Our study provides strong evidence of the value of UAS for HTP applied to large wheat breeding nurseries. Further work is needed to investigate the strength of the relationship between remotely sensed derived plant phenological traits and the wheat biophysical properties collected in the field such as plant height, biomass, and yield. With the overall vision of integrating multiple measurements extracted from UAS (plant height, ground cover, etc.) with plant growth simulations to maximize the biological utility of the estimated phenotypes new avenues will be opened to breeders for predicting yield. Moreover, the development of new sensors and imaging systems undoubtedly will continue to improve our ability to phenotype very large experiments or breeding nurseries. When combined with genomic and physiological modeling, the rapid, low-cost evaluation of large field trials in plant breeding with UAS platforms has the potential to greatly accelerate the breeding process through more accurate selections on larger populations.

## Abbreviations

BER: band equivalent reflectance
CIMMYT: International Maize and Wheat Improvement Center
DEM: digital elevation model
DN: digital number
DPP: digital photo professional
ENDVI: enhanced normalized difference vegetation index
FWHM: full width at half maximum
GCP: ground control points
GIPVI: infrared percentage vegetation index
GNDVI: green normalized difference vegetation index
GSD: ground sampling distance
HTP: high-throughput phenotyping
LAI: leaf area index
NDVI: normalized difference vegetation index
NIR: near infrared
RENDVI: red-edge normalized difference vegetation index
RMSE: root mean square error
SIFT: scale-invariant feature transform
UAS: unmanned aerial system
UAV: unmanned aerial vehicle
VI: vegetation index
